# Genomic analysis of *Brucella* isolates from animals and humans, Türkiye, 2010 to 2020

**DOI:** 10.2807/1560-7917.ES.2024.29.38.2400105

**Published:** 2024-09-19

**Authors:** Kadir Akar, Hanka Brangsch, Tariq Jamil, Gülseren Yıldız Öz, Emin Ayhan Baklan, Buket Eroğlu, Eray Atıl, Sevil Erdenlig Gürbilek, Oktay Keskin, Osman Yaşar Tel, Ayfer Güllü Yücetepe, Vassilios Sandalakis, Evridiki Boukouvala, Anna Psaroulaki, Ashraf A Abd El Tawab, Falk Melzer, Mathias W Pletz, Heinrich Neubauer, Gamal Wareth

**Affiliations:** 1 Department of Microbiology, Faculty of Veterinary Medicine, Van Yuzuncu Yıl University, Van, Türkiye; 2 Institute of Bacterial Infections and Zoonoses, Friedrich-Loeffler-Institut, Jena, Germany; 3 National Reference Laboratory (NRL) for Brucellosis, Pendik Veterinary Control Institute, Istanbul, Türkiye; 4 Microbiology Department, Faculty of Veterinary Medicine, University of Harran, Şanlıurfa, Türkiye; 5 Department of Clinical Microbiology and Microbial Pathogenesis, School of Medicine, University of Crete, Heraklion, Greece; 6 Hellenic Agricultural Organization-DIMITRA, Veterinary Research Institute, Thessaloniki, Greece; 7 Department of Bacteriology, Immunology and Mycology, Faculty of Veterinary Medicine, Benha University, Moshtohor, Toukh, Egypt; 8 Institute of Infectious Diseases and Infection Control, Jena University Hospital, Jena, Germany

**Keywords:** *Brucella melitensis*, *Brucella abortus*, WGS, cgSNP, One-Health, Türkiye, zoonotic infections, brucellosis, *Brucella*, biosafety

## Abstract

**Background:**

Brucellosis is a bacterial zoonosis causing severe illness in humans and animals and leading to economic losses in the livestock production in Türkiye and other endemic countries.

**Aim:**

We aimed at investigating genomic differences of *Brucella* isolates from animals and humans in Türkiye.

**Methods:**

We used whole genome sequencing (WGS) to assess the genetic diversity of *Brucella* isolates from 41 provinces in Türkiye and compared with isolates from other countries. We applied allele-based typing and core genome single nucleotide polymorphism (cgSNP) determination.

**Results:**

Of the 106 Turkish *Brucella* isolates included, 57 were *B. abortus* and 49 were *B. melitensis*. One *B. melitensis* and two *B. abortus* isolates were identified as vaccine strains. Most (n = 55) *B. abortus* isolates clustered in three major branches, with no spatial discernible pattern. Of the *B. melitensis* isolates*,* 48 were assigned to the Eastern Mediterranean lineage with no discernible patterns between host species, location and sampling date. The Turkish isolates clustered with isolates from neighbouring countries such as Greece and Syria, but some also with isolates from human patients in European countries, like Germany, Norway and Sweden, suggesting that the source may be travel-related.

**Conclusion:**

Several *B. melitensis* and *B. abortus* lineages are circulating in Türkiye. To decrease the prevalence and prevent brucellosis in animals and humans, stricter control measures are needed, particularly in areas where humans and animals have close contact. Furthermore, illegal transportation of animals across borders should be more closely controlled and regulated.

**Key public health message**

**What did you want to address in this study and why?**

Brucellosis is a chronic disease of humans and animals, caused by bacteria of the genus *Brucella*. The disease is common in Türkiye. We sequenced *Brucella* isolates to analyse the similarity between human and ruminant isolates in Türkiye and compared them with isolates from other countries.

**What have we learnt from this study?**

Turkish *Brucella* isolates were similar to isolates from neighbouring countries such as Greece and close to isolates from patients from Germany, Norway and Sweden, indicating that the infections were travel-associated. Open borders and uncontrolled transboundary movement of animals and non-compliance with prevention and control programmes have an impact on persistence of brucellosis in Türkiye.

**What are the implications of your findings for public health?**

Stringent control programmes in animals and pasteurisation of milk are essential measures in preventing brucellosis in humans and animals. In most European countries, brucellosis is predominantly diagnosed in travellers visiting areas where the disease is common, such as Türkiye. Consumption of contaminated food is the most common source of infection, and people should avoid consuming raw dairy and meat products.

## Introduction

Brucellae are Gram-negative facultative intracellular bacteria that cause brucellosis in many domestic and wild animal species and humans [1]. In animals, brucellosis presents predominantly with reproductive symptoms, arthritis and decreased milk production, and in humans, as an acute or chronic febrile illness with malaise, fatigue and arthritis. Brucellosis is seldom fatal, but chronic infection is often debilitating, and severe complications may arise. Transmission between animals occurs most often via mating with infected animals and from animals to humans via direct or indirect contact with contaminated tissues and fluids (e.g. placenta, blood, aborted tissues, uterine discharge) or consumption of contaminated unpasteurised milk and milk products [1].

Due to the low infectious dose, possibilities for airborne transmission, the chronic disease and difficult treatment of the disease, some pathogenic *Brucella* spp. have been classified as category B biological threats by the National Centre for Emerging and Zoonotic Infectious Diseases of the United States (US) [2]. Knowledge of the epidemiology of the disease, including prevalence of *Brucella* species in endemic countries, is vital for establishing and implementing reliable and effective control measures against brucellosis, especially within the One Health context [3].

Brucellosis is endemic in several countries worldwide, particularly in the Middle East, Mediterranean region, Africa and Central and South America [4,5]. The estimated global annual incidence in humans is 2.1 million cases [6]. Diagnosis of brucellosis is challenging, and the disease has a considerable negative impact on the health and income of livestock farmers worldwide, especially where surveillance and vaccination programmes are lacking [1,7]. In endemic countries, these bacteria pose a considerable public health risk to humans, arising mainly from the consumption of contaminated unpasteurised milk or milk products. In addition, travel-associated cases are recorded in brucellosis-free countries [4,8,9].

In Türkiye, brucellosis is a notifiable endemic disease in animals and humans, associated with major economic losses in the livestock industry due to abortions, reduced fertility, obligatory slaughtering of seropositive animals and vaccination. Up-to-date estimates of the true prevalence for brucellosis are lacking in Türkiye, because of lack of awareness and reluctance among farmers to notify due to low economic compensation. Before introduction of vaccination programmes in 2012, the prevalence was estimated to 7.8% in cattle and 22.5% in sheep [10]. In a study of brucellosis among residents in rural areas of Türkiye (2003–2017), seropositivity to *Brucella* could be as high as 11.9% [11]. In a study conducted in the Ordu province, 7.8% of serum samples from people in risk groups, such as veterinarians, farmers and butchers, were serologically positive [12]. In 2019, 10,244 human brucellosis cases were notified, which was an increase from 4,173 cases in 2015 [13].

Brucellosis is endemic in domestic animals in countries neighbouring Türkiye, e.g. Greece and Bulgaria [14], Azerbaijan [15], Iran, Syria and Iraq [16], whereas most European Union/European Economic Area (EU/EEA) countries are free of brucellosis. In 2022, 198 cases of brucellosis were notified in humans in the EU/EEA, most of them were infected in endemic countries [17]. To eradicate brucellosis, stringent preventive and control measures are needed, e.g. identification of infected herds, control of animal movements and strict quarantine and sanctions for animals moved if transported uncontrolled. Although animal vaccination, quarantine and compensated slaughter protocols are implemented, uncontrolled and illegal livestock movements and limited veterinary support services are among several factors contributing to the persistence of the disease in Türkiye [10]. In addition, outbreaks in animals and humans should be traced back, contained and the source identified by applying epidemiological and molecular methods [18,19].

*Brucella melitensis* and *B. abortus* are the main *Brucella* species circulating in Türkiye. Previously, the connection between *B. melitensis* isolates from different geographical regions in Türkiye was investigated using multiple-locus variable number tandem repeat analysis (MLVA) and classical multilocus sequence typing (MLST) [13,20]. Since *Brucella* genomes are highly clonal, techniques with higher discriminatory power, e.g. core genome (cg) MLST (cgMLST) and single nucleotide polymorphism (SNP) analysis based on whole genome sequencing (WGS), are needed for detailed analysis [21-25].

We aimed to apply WGS-based cgSNP analysis on *B. abortus* and *B. melitensis* isolates from animals and humans in Türkiye to investigate the diversity and genetic relationships of the prevailing lineages in the country and compare with isolates from other countries.

## Methods

### Origin and phenotypic testing of *Brucella* isolates

A total of 106 *Brucella* isolates from cattle (n = 39), buffaloes (n = 6), sheep (n = 28), goats (n = 26) and humans (n = 7) were selected from the isolate collection of Pendik Veterinary Control Institute (PVCI, Istanbul, Türkiye) and the Department of Veterinary Microbiology of Harran University (Şanlıurfa, Türkiye). The isolates originated from samples taken nationwide between 2010 and 2020. Metadata of all isolates, including host species, location and sampling date, are presented in Supplementary Table S1. The isolates were characterised based on colony morphology, CO_2_ requirement, H_2_S production, oxidase, catalase and urease activity. Further, growth characteristics in a medium containing basic fuchsin (20 μg/mL), thionine (20 μg/mL), penicillin (5 IU/mL), streptomycin (2.5 μg/mL) and i-erythritol (1 mg/mL) and susceptibility to *Brucella* phages at routine test dilution (RTD) and 10^4 ^× RTD by Tbilisi, R/C and Izatnagar phages were analysed as previously described [26]. After these phenotypic tests, the isolates were submitted to the Institute of Bacterial Infections and Zoonoses (IBIZ) of Fredrich-Loeffler-Institut, Jena, Germany for confirmation and sequencing. A Nagoya agreement for receiving and using biological material from Türkiye is not required. Species identification of the isolates was performed using AMOS-PCR [27].

### Whole genome sequencing

Genomic DNA was extracted with the High Pure PCR Template Preparation Kit (Roche, Basel, Switzerland) according to the manufacturer's instructions. Nextera XT DNA Library Preparation Kit (Illumina, San Diego, US) was used for library preparation, and whole genome sequencing was performed on a MiSeq system using v3 chemistry (Illumina) for 2 × 300 bp long reads.

#### Quality control and assembly

Raw sequencing data were controlled and assembled as described before [24] using FASTQC v0.11.7 [28], Kraken2 v2.0.7_beta [29], Shovill v.1.0.4 (https://github.com/tseemann/shovill) and QUAST v5.0.2 [30]. Following the assembly, the species identification of the isolates was verified by screening the contigs with Kraken2.

#### Genotyping

Firstly, the genomic diversity of the isolates was assessed by in-silico MLST, using the tool mlst v2.19.0 (https://github.com/tseemann/mlst) and the MLST-9 scheme [31] of the PubMLST website [32]. For in-depth analysis, SNPs of the isolates were analysed using Snippy v.4.6.0 (https://github.com/tseemann/snippy) with subsequent maximum likelihood analysis of the cgSNP alignment by RAxML v8.2.12 [33]. The reference genomes were *B. abortus* 2308, (GCF_000054005.1), *B. melitensis* 16M (GCF_000007125.1) and *B. melitensis* M28 (GCF_000192725.1).

We screened the short-read archive (SRA) of the National Center for Biotechnology Information (NCBI; https://www.ncbi.nlm.nih.gov/) (accessed on 4 April 2023) for Illumina data of *B. abortus* and *B. melitensis* isolates from Türkiye and other countries. The datasets were downloaded, and their quality was checked for the raw read data. The data of the downloaded genomes, including accession numbers, *Brucella* spp., host species, year and country of isolation, are presented in Supplementary Table S2. We assessed SNP differences using snp-dists v0.7.0 (https://github.com/tseemann/snp-dists) and the hclust function in R (R Core Team 2022) [34].

### Visualisation

Microreact [35] was used for visualising the epidemiological data and the phylogenetic trees. A map was also created using Microreact with the implemented Maps Mapbox (https://www.mapbox.com/about/maps) and OpenStreetMap (https://www.openstreetmap.org/about).

## Results

### Descriptive epidemiological findings

**Figure 1:**
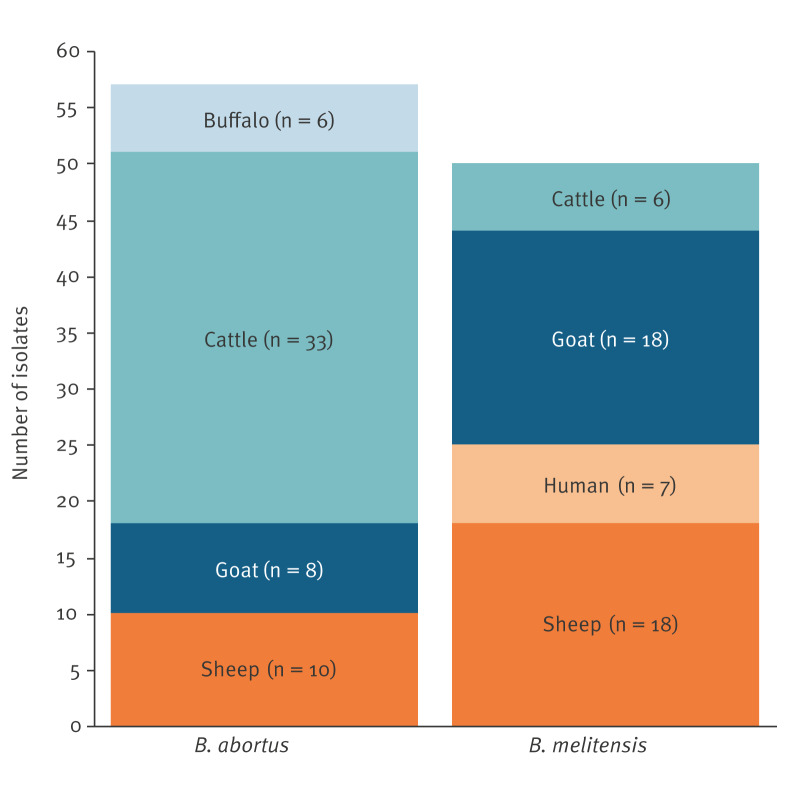
Isolates of *Brucella abortus* and *Brucella melitensis*, by host species, Türkiye, 2010–2020 (n = 106)

**Figure 2:**
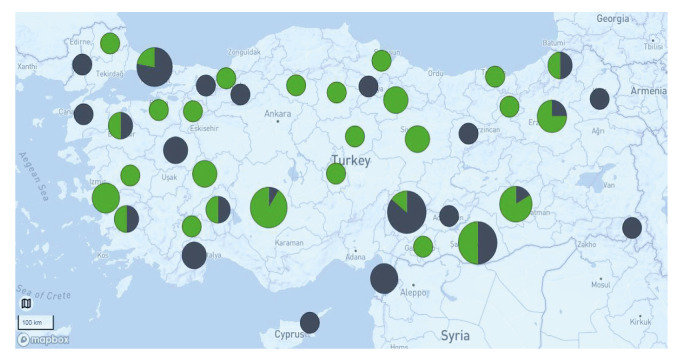
Geographical distribution of *Brucella abortus* (n = 57) and *Brucella melitensis* (n = 49) isolates, Türkiye, 2010–2020

Of the 106 *Brucella* isolates included in this study, 49 were identified as *B. melitensis* and 57 as *B. abortus*. In agreement with the known host preferences of both species, the *B. abortus* isolates were primarily from cattle (n = 33) and small ruminants (n = 18) (Figure 1) and *B. melitensis* isolates from small ruminants (n = 36), although six *B. melitensis* isolates were from cattle. The seven isolates from human patients were *B. melitensis*. Only *B. abortus* was isolated from buffaloes. The isolates originated from 41 of the 81 provinces of Türkiye (Figure 2). From most of these provinces, only a single isolate was available. However, from Kahramanmaraş and Şanlıurfa provinces in southeastern Türkiye, 14 isolates were included. In Kahramanmaraş, several small-scale dairies sell goat milk products. No spatial pattern was observed in the distribution of the two *Brucella* species across the country. The human isolates were from three provinces in southern Türkiye and Istanbul.

*B: Brucella*.

*Brucella abortus* isolates are marked in green, *Brucella melitensis* isolates in black. The size of the circle is relative to the number of isolates from the respective province.

The map was created using Mapbox (www.mapbox.com/about/maps) and OpenStreetMap (www.openstreetmap.org/about).

### Biotypes and sequence types of Turkish isolates

Biovar (bv) 3 was identified in 40 *B. melitensis* isolates, bv1 in seven, bv2 in one and Rev 1 vaccine strain in one isolate. Most (50/57) of the *B. abortus* isolates belonged to bv3, three to bv1, two were S19 vaccine strains, one was bv2 and one bv9.

Draft genomes obtained by de novo assembly comprised 15–47 contigs adding up to genomes of 3,281,492–3,293,217 bp for *B. melitensis* and 3,244,460–3,304,060 bp for *B. abortus* isolates. The guanine and cytosine (GC) content of all isolates varied between 57.13 and 57.28%. The genomic data of the sequenced isolates are presented in Supplementary Table S3.

Four different sequence types (STs) were identified among the *B. abortus* isolates: ST1 (n = 1), ST2 (n = 36), ST3 (n = 18) and ST5 (n = 2). Sequence types ST2 and ST3 were identified in *B. abortus* isolates from all four animal species and from multiple provinces without any discernible pattern whereas ST1 was only found in cattle from southeast (Şanlıurfa) in 2017. The two ST5 isolates characterised as *B. abortus* S19 vaccine strains by phenotyping and PCR were from a goat and a sheep from provinces in western and eastern Türkiye and isolated in 2011 and 2014, respectively.

**Figure 3:**
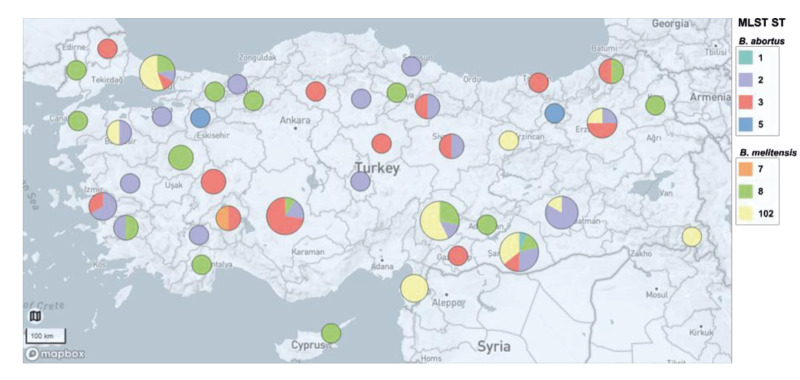
Geographical distribution of multilocus sequence types of *Brucella abortus* (n = 57) and *Brucella melitensis* (n = 49) isolates, Türkiye, 2010–2020

MLST: multilocus sequence typing; ST: sequence type.

Three STs were identified among the 49 *B. melitensis* isolates: ST7 (n = 1), ST8 (n = 22) and ST102 (n = 26). The ST7 isolate, TUR-S-Ispar-2019, from a sheep from Isparta in southwestern Türkiye, was identified as a Rev 1 vaccine strain. The ST8 and ST102 isolates were from goats, sheep, humans and cattle from different provinces, with no discernible preference for host, time or place. The geographical distribution of different STs is shown in Figure 3.

### Phylogeny and international context of *Brucella abortus* isolates

**Figure 4:**
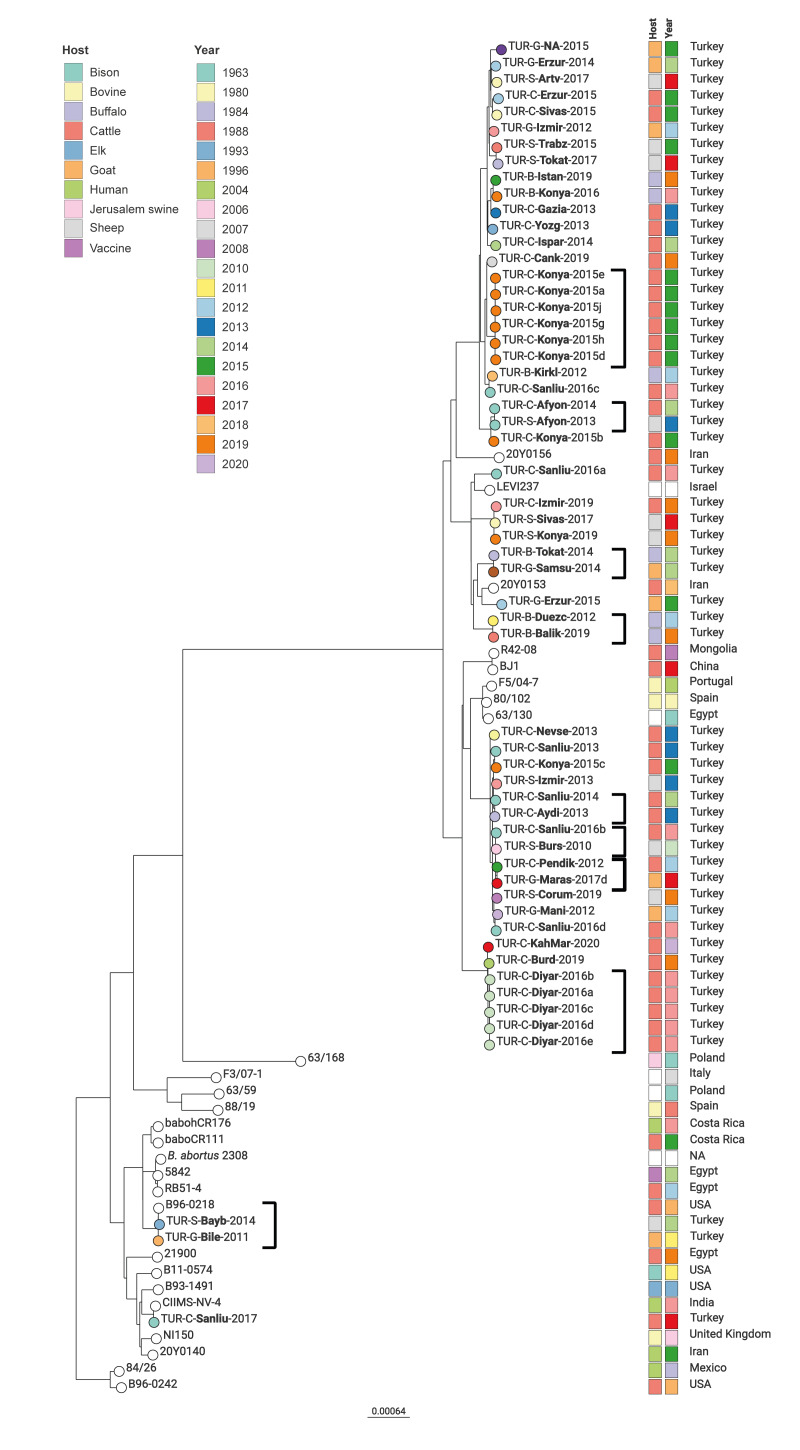
Phylogenetic tree based on single nucleotide polymorphism analysis of *Brucella abortus* isolates, Türkiye, 2010–2020 (n = 57), compared with isolates from the National Center for Biotechnology Information (NCBI) short read archive (SRA), 1963–2020 (n = 24; accessed on 4 April 2023)

In addition to the Turkish isolates, we included the reference strain *B. abortus* 2308 and 24 isolates from the Mediterranean region (Egypt, Israel, Italy, Spain), other European countries (Poland, Portugal, United Kingdom), North America (US, Mexico) and Asia (China, India, Iraq, Mongolia) in the phylogenetic analysis (Figure 4). The Turkish isolates formed several clusters on one branch of the phylogenetic tree, with no association between sampling time, place or the host of an isolate, as isolates could be from samples taken up to 9 years apart. However, within some clusters, the distances were > 20 SNPs.

B: bison; C: cattle; G: goat; S: sheep; TUR: Türkiye.

The blocks to the right show the host species, year and the country of origin. The leaf colours of the isolates investigated here indicate the Turkish province of isolation, and black brackets show isolates with ≤ 5 SNPs distances. The bar at the bottom indicates base changes per site.

Of the eight cattle isolates from Konya from 2015, six formed a cluster with ≤ 4 SNPs distance (one outbreak), while the other two isolates displayed 81–83 and 281–283 cgSNPs differences to this cluster. One of these diverging isolates (TUR-C-Konya-2015c), isolated in 2015, had a 7 SNPs distance to isolate TUR-C-Sanliu-2013, which was from cattle sampled in 2013 in another province.

Additional eight Turkish clusters with ≤ 5 SNPs distances were identified, with isolates from different years and provinces. Eight isolates from Iran, Israel, Egypt, Spain and Portugal were in the same branch of the tree, between clusters of Turkish isolates. Isolates TUR-S-Bayb-2014 and TUR-G-Bile-2011, from a sheep in eastern Türkiye and a goat in western Türkiye and identified as vaccine strain S19, differed by 1–4 SNPs from an isolate from cattle in the US, indicating that the vaccine strain might cause brucellosis.

A Turkish *B. abortus* isolate, TUR-C-Sanliu-2017, from cattle in Şanlıurfa, was similar to CIMS-NV-4, isolated from a sample from an Indian person 1 year earlier. These isolates differed by 6 cgSNPs. Also, five cattle isolates from southeast (Diyarbakir) from 2016 had a 7–8 SNPs distance to a 2019 isolate from southwest (Burdur) and to a 2020 isolate from Kahramanmaraş.

### Phylogeny and international context of *Brucella melitensis* isolates

*Brucella melitensis* 16M, used as a reference genome, and at least one representative strain from each of the four main lineages and sublineages of the Eastern Mediterranean lineage was used, including the recently found novel sublineage, as described previously [24]. We analysed isolates of the American and the East Mediterranean lineages separately.

**Figure 5:**
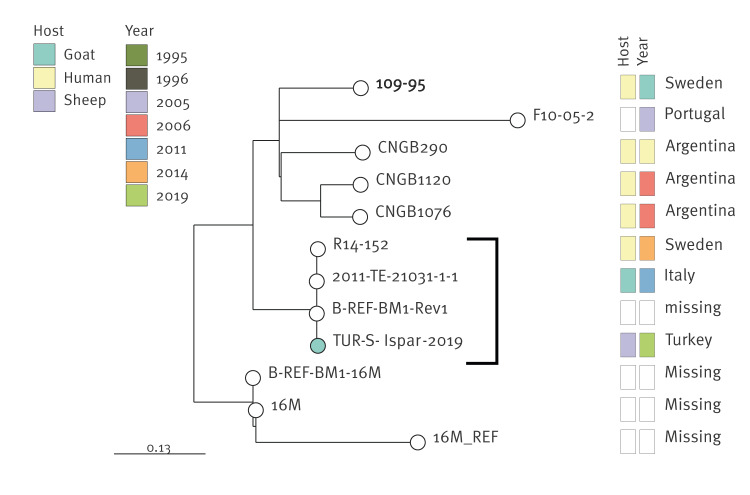
Maximum likelihood tree based on core genome single nucleotide polymorphism alignment of the American lineage isolates of *Brucella melitensis,* Türkiye, 2019 and other countries, 1995–2019 (n = 12)

Only one Turkish *B. melitensis* isolate (TUR-S-Ispar-2019), identified as Rev 1 vaccine strain, belonged to the American lineage and differed by 2 cgSNPs from the vaccine strain (Figure 5). Also, three other isolates clustered with TUR-S-Ispar-2019 and *B. melitensis* Rev 1: an isolate from a human patient from Sweden, a goat isolate from Italy and an isolate with no information about the country of isolation or host species. These can, thus, be assumed to be of vaccine origin.

The boxes to the right show the isolation host species and isolation year and the country of origin. The isolate from the Turkish province (Isparta) is marked blue. The square brackets show isolates that differ with ≤ 5 SNPs. The bar at the bottom indicates base changes per site.

**Figure 6:**
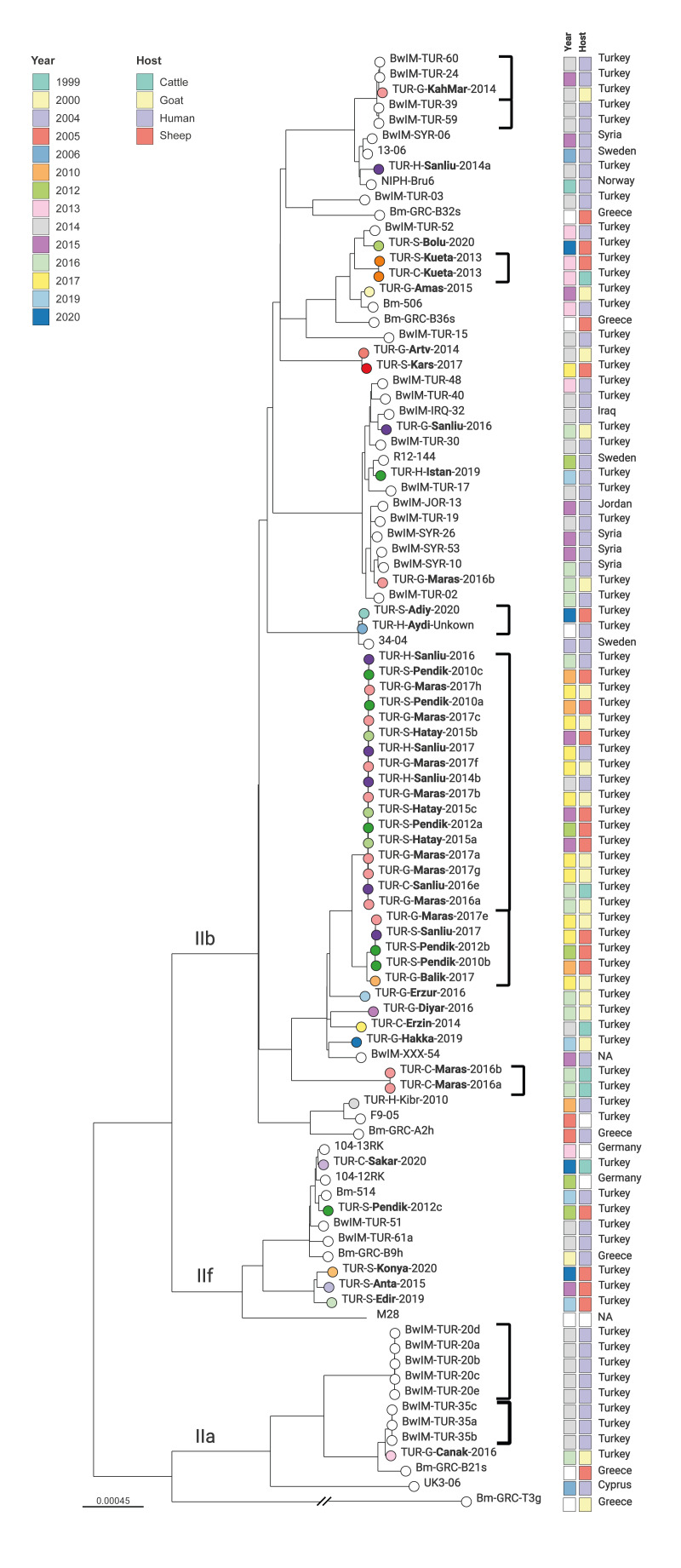
Maximum likelihood tree based on core genome single nucleotide polymorphism alignment of *Brucella melitensis* isolates of the Eastern Mediterranean lineage, Türkiye, 2010–2020 (n = 48) and isolates from Cyprus, Germany, Greece, Iraq, Jordan, Norway, Sweden, Syria, 1999–2019 (n = 47)

Most Turkish isolates (48/49) belonged to the Eastern Mediterranean lineage genotypes IIa (n = 1), IIb (n = 42), and IIf (n = 5). TUR-G-Canak-2016, of genotype IIa, was isolated from a goat in the province Çanakkale, located on the northwestern border of Türkiye. It differed by 13 SNPs from isolates from three human patients sampled in 2014 and by 44 SNPs from a Greek sheep isolate (Figure 6). The five isolates (TUR-C-Sakar-2020, TUR-S-Pendik-2012c, TUR-S-Konya-2020, TUR-S-Anta-2015 and TUR-S-Edir-2019) of the IIf lineage were mostly from sheep and isolated between 2012 and 2020. Isolates TUR-S-Pendik-2012c and TUR-C-Sakar-2020 had 18–13 SNPs distance to isolates from human patients from Türkiye and Germany (Bm-514 and 104-13RK). The human patients were sampled 7 years earlier than the animals.

The boxes to the right show the host species, isolation year and the country of origin. Leaf colours of the here investigated isolates indicate Turkish province of isolation and black brackets show isolates that differ by ≤ 5  SNPs. Sublineages (IIa, IIb, and IIf) are indicated. The bar at the bottom indicates base changes per site in the alignment.

Most Turkish isolates belonged to lineage IIb and clustered with isolates from Greece, Iraq, Jordan, Norway, Sweden, Syria and Türkiye, isolated primarily from human patients. For example, TUR-H-Sanliu-2014a with 22 cgSNPs distance to NIPH-Bru6 was isolated from a human patient in Norway in 1999. The largest cluster was found among isolates in the IIb lineage, comprising 17 isolates from goats, sheep, cattle and humans from four provinces in Türkiye (Istanbul, Şanlıurfa, Kahramanmaraş and Hatay) 2010–2017. Except for Istanbul, the other three provinces are located near the border with Syria. The three human isolates in this cluster were from Şanlıurfa (TUR-H-Sanliu-2014b, TUR-H-Sanliu-2016, TUR-H-Sanliu-2017) from 2014, 2016 and 2017. The only other isolate from the same province in this cluster was from cattle (TUR-C-Sanliu-2016e), whereas the other isolates were retrieved from sheep and goats (n = 13) in other provinces. The cattle isolate (TUR-C-Sanliu-2016e) and the human isolates (TUR-H-Sanliu-2014b, TUR-H-Sanliu-2016, TUR-H-Sanliu-2017) displayed no SNP differences. In the same branch, there was a smaller cluster with sheep and goat isolates (TUR-G-Maras2017e, TUR-S-Sanliu-2017, TUR-S-Pendik-2010b, TUR-S-Pendik-2012b, TUR-G-Balik-2017).

A sheep isolate, TUR-S-Adiy-2020, from Adiyaman (southeast Türkiye) was close (≤ 5 SNPs) to an isolate from a human patient (TUR-H-Aydi-Unknown) from Aydin (west Türkiye). These two isolates had a 15–17 SNPs distance to isolate 34–04 from a human patient from Sweden. Further, a Turkish goat isolate (TUR-G-KahMar-2014) from 2014 was highly similar (5 SNPs difference) to two Turkish human isolates (BwIM-TUR-60, isolated in 2014 and BwIM-TUR-24, isolated in 2013). One isolate from sheep (TUR-S-Kueta-2013) and cattle (TUR-C-Kueta-2013) from Kutahya (western Türkiye), from 2013 clustered together with distance of 1 SNP.

## Discussion

*Brucella* spp. have been classically typed by identification of biovars with phenotypic methods, including biochemical tests and phage sensitivity. In previous genotyping studies of Turkish *B. melitensis* isolates with MLVA and MLST, identical genotypes were identified in various geographical locations [20,36]. However, MLVA-based results should be interpreted with caution due to the impact of homoplasy on the target repeats [37,38], whereas SNP analyses allow more accurate differentiation of isolates of the same species [23,24].

Three of the 106 isolates in this study were identified as vaccine strains, either *B. abortus* S19 or *B. melitensis* Rev 1. The production of *B. abortus* S19 and *B. melitensis* Rev 1 vaccines started in 1960 and 1969, respectively [39]. Both vaccines use live-attenuated strains, which may cause abortions in pregnant animals [40]. The vaccine strains can cause disease in humans as they can enter the mammary glands of the vaccinated animals, thus shed in the milk. Also, the vaccine-induced antibodies may interfere with serodiagnosis of vaccinated animals. Improper use of vaccines, e.g. vaccination of pregnant animals, might spread the disease due to insufficient implementation of control and eradication programmes and a lack of health education of farmers.

Most Turkish livestock farmers have goats and sheep. These animals are often kept in mixed herds, particularly in central, east and southeast Anatolia [41], which explains the genetic similarity of sheep and goat *B. melitensis* isolates. Except for the intensive livestock farming, animals are often in direct contact with humans and graze freely, where contact with other possibly infected animals can hardly be prevented [41,42]. Goat farms in Türkiye are mainly in the southern provinces and along the Mediterranean Sea coast [41], which was also reflected in the number of *B. melitensis* isolates from these provinces i.e. the largest *B. melitensis* cluster comprising goat, sheep and human isolates included mostly isolates from the three southern provinces (Kahramanmaraş, Hatay, Şanlıurfa). Nomads often have goats and sheep, which generally are not vaccinated against brucellosis, and usually move between the eastern and the southeastern provinces depending on the weather conditions [41]. This movement of animals could promote the spread of brucellosis [43]. However, although the isolates investigated in this study came from 41 provinces, data from other regions where goat breeding is intensive, e.g. Mersin and Adana provinces, are lacking [44], which hampers further epidemiological investigations.

Most *B. abortus* isolates belonged to a distinct lineage comprising Iranian and Israeli strains, indicating that the prevailing *B. abortus* strains have been endemic in this region for a long time, and that there is frequent contact or movement of animals in the bordering region. The high diversity between the isolates might be explained by the fact that there are only a few large-scale cattle farms in Türkiye, and almost 50% of dairy farms have less than six animals [45].

Some *B. melitensis* isolates shared high similarity to German, Swedish and Norwegian strains, suggesting that these strains may be travel-related [9]. Although the distances between the isolates from animals and the European human patients were > 10 SNPs, they may share the same ancestry. This time span may allow for genome mutations in the bacterium. However, as there was no geographical clustering in Türkiye and we had scarce metadata, we cannot pinpoint the location of the human infections. The same applies to human isolates from Türkiye included in this study, which were highly similar to the animal isolates.

The production of cheese and cream in family businesses, in particular, can be seen as a risk factor, as the products are placed on the market without any controls [46,47]. There are few centres in Türkiye where most dairy products are manufactured. For instance, butter made from goat and sheep milk in Şanlıurfa and goat milk ice cream produced in Kahramanmaraş are famous and are distributed to major cities across the country [10]. This might explain the remarkable similarity between sheep and goat isolates in both provinces with human isolates in other cities. During the annual Islamic festival of Eid al-Adha ‘the biggest Islamic festival of sacrifice’, the shipment of animals from various regions of Türkiye poses a considerable risk of spreading brucellosis. Animals are often butchered in residential areas and on public thoroughfares instead of designated slaughterhouses, posing a considerable public health hazard, particularly in rural and small-town settings. Some of these animals are not slaughtered and are instead kept for the following year, allowing for potential breeding and the transmission of the disease between regions. This practice necessitates thoughtful consideration and appropriate measures to prevent the spread of pathogens. The exchange of animals between farms and animal markets was found a major driver of brucellosis spread and even of human brucellosis cases, particularly when the markets are not regulated, and public health guidelines are not followed [48]. Overall, it can be expected that the trade with animals within Türkiye, e.g. on animal markets or import of animals for breeding purposes, is one of the main drivers for brucellosis transmission, promoting the spread of different *Brucella* lineages throughout the country.

The Turkish isolates of *B. melitensis* were similar to isolates from neighbouring countries such as Greece and Syria which indicates that there is a cross-border transmission between the countries, although no recent events could be detected, as SNP differences were too large for drawing definite conclusions. Illegal and uncontrolled animal crossing might promote the spread of the bacterium within regions, contributing to the persistence of brucellosis in border provinces [10]. However, we consider that these lineages have been circulating in Türkiye for a long time and that they have diverged from a presumed common ancestor over a long period of time, resulting in diverse genotypes that differ in hundreds of SNPs.

In the 1980s, a programme was launched to control and eradicate brucellosis. Although a new national brucellosis control and eradication programme using mass vaccination strategy with conjunctively applied vaccines was implemented in Türkiye in 2012 [49,50], it has not resulted in the desired control of the disease. The main reason for this failure is low vaccination coverage driven by the fear of acquiring infection from *Brucella* vaccines, poor logistics in vaccine transport in some areas and general unwillingness to use these vaccines. Farmers may be unwilling to suspect brucellosis to avoid economic losses when animals positive for *Brucella* are slaughtered. Further, the prevailing nomadic livestock husbandry and the lack of preventive veterinary measures will allow the transmission of *Brucella* species between regions [41,50,51].

Brucellosis is endemic in the Balkans and the Middle East. Syria has one of the highest numbers of notified human cases of brucellosis in the world [5,52]. Open borders and uncontrolled transboundary movement of animals play a significant role in the transmission and persistence of brucellosis in Türkiye. In the southeastern region of Türkiye and neighbouring countries, most brucellosis cases in humans are linked to consumption of unpasteurised milk or cream cheese [47,53-56]. In addition, occupational cases originate from areas with intensive animal husbandry where human-animal interaction is frequent. Preventive measures and precautions when handling infected animals are crucial for people working in this field. A modelling study from Jordan showed that the preventive measures with the greatest impact included controlling animal markets, implementing of a strict test and cull policy and applying a One Health approach combining measures of veterinary and public health sectors [48]. The same impact of these measures can be assumed for Türkiye.

The main challenges faced by the current study are the need for up-to-date incidence and prevalence data on brucellosis in the country, including more metadata of collected isolates. This can be achieved by increasing awareness and providing fair compensation for infected animals, to promote notifications. According to the regulations in Türkiye, all *Brucella* isolates obtained from miscarriages in animals need to be biotyped and stored by Pendik Veterinary Institute. However, complete metadata are not included in the isolate collection; only sampling year, host species, location of the isolate are recorded. Furthermore, official import and export data are not accessible, and it is difficult to retrieve this kind of information.

## Conclusion

Brucellosis is a widespread disease in Türkiye and has a significant impact on public and animal health. Several lineages of *B. melitensis* and *B. abortus* are circulating in Türkiye. The majority of *B. melitensis* isolates belong to the Eastern Mediterranean lineage, with some sharing a high similarity to isolates from human cases in Europe, indicating potential travel-related transmission [8,9]. Turkish *B. abortus* isolates are closely related to isolates from neighbouring Asian countries, highlighting the role of animal movement in the spread of infection across borders which also suggests the longstanding prevalence of *B. abortus* in this region. Strengthening surveillance of brucellosis, including reporting and data collection and implementing strict control and preventive measures, is essential to reduce the burden of the disease. It is recommended to enforce standard rules in the production of milk and dairy products, and to closely monitor and regulate the illegal transportation of animals across borders. Increasing the number of isolates for analysis can provide more accurate information on the epidemiological distribution of the disease.

## Supplementary Data

46000 Supplementary Material

## References

[1] Qureshi KA Parvez A Fahmy NA Abdel Hady BH Kumar S Ganguly A Brucellosis: epidemiology, pathogenesis, diagnosis and treatment-a comprehensive review. Ann Med. 2023;55(2):2295398. 10.1080/07853890.2023.2295398 38165919 PMC10769134

[2] Pappas G Panagopoulou P Christou L Akritidis N. Brucella as a biological weapon. Cell Mol Life Sci. 2006;63(19-20):2229-36. 10.1007/s00018-006-6311-4 16964579 PMC11136069

[3] De Massis F Zilli K Di Donato G Nuvoloni R Pelini S Sacchini L Distribution of Brucella field strains isolated from livestock, wildlife populations, and humans in Italy from 2007 to 2015. PLoS One. 2019;14(3):e0213689. 10.1371/journal.pone.0213689 30901346 PMC6430384

[4] Pappas G Papadimitriou P Akritidis N Christou L Tsianos EV. The new global map of human brucellosis. Lancet Infect Dis. 2006;6(2):91-9. 10.1016/S1473-3099(06)70382-6 16439329

[5] Bagheri Nejad R Krecek RC Khalaf OH Hailat N Arenas-Gamboa AM. Brucellosis in the Middle East: Current situation and a pathway forward. PLoS Negl Trop Dis. 2020;14(5):e0008071. 10.1371/journal.pntd.0008071 32437346 PMC7241688

[6] Laine CG Johnson VE Scott HM Arenas-Gamboa AM. Global estimate of human brucellosis incidence. Emerg Infect Dis. 2023;29(9):1789-97. 10.3201/eid2909.230052 37610167 PMC10461652

[7] Elrashedy A Gaafar M Mousa W Nayel M Salama A Zaghawa A Immune response and recent advances in diagnosis and control of brucellosis. Ger J Vet Res. 2022;2(1):10-24. 10.51585/gjvr.2022.1.0033

[8] Johansen TB Scheffer L Jensen VK Bohlin J Feruglio SL. Whole-genome sequencing and antimicrobial resistance in Brucella melitensis from a Norwegian perspective. Sci Rep. 2018;8(1):8538. 10.1038/s41598-018-26906-3 29867163 PMC5986768

[9] Georgi E Walter MC Pfalzgraf MT Northoff BH Holdt LM Scholz HC Whole genome sequencing of Brucella melitensis isolated from 57 patients in Germany reveals high diversity in strains from Middle East. PLoS One. 2017;12(4):e0175425. 10.1371/journal.pone.0175425 28388689 PMC5384748

[10] Yumuk Z O’Callaghan D. Brucellosis in Turkey -- an overview. Int J Infect Dis. 2012;16(4):e228-35. 10.1016/j.ijid.2011.12.011 22333223

[11] Vancelik S Guraksin A Ayyildiz A. Seroprevalence of human brucellosis in rural endemic areas in eastern Turkey. Trop Doct. 2008;38(1):42-3. 10.1258/td.2007.060004 18302867

[12] Cetinkol Y Enginyurt Ö Çelebi B Yıldırım AA Çankaya S Aktepe OC. Investigation of zoonotic infections in risk groups in Ordu University Hospital, Turkey. Niger J Clin Pract. 2017;20(1):6-11. 10.4103/1119-3077.181395 27958239

[13] Akar K Tatar F Schmoock G Wareth G Neubauer H Erganis O. Tracking the diversity and Mediterranean lineage of Brucella melitensis isolates from different animal species in Turkey using MLVA-16 genotyping. Ger J Vet Res. 2022;2(1):25-30. 10.51585/gjvr.2022.1.0037

[14] Cross AR Baldwin VM Roy S Essex-Lopresti AE Prior JL Harmer NJ. Zoonoses under our noses. Microbes Infect. 2019;21(1):10-9. 10.1016/j.micinf.2018.06.001 29913297 PMC6386771

[15] Khatibi M Abdulaliyev G Azimov A Ismailova R Ibrahimov S Shikhiyev M Working towards development of a sustainable brucellosis control programme, the Azerbaijan example. Res Vet Sci. 2021;137:252-61. 10.1016/j.rvsc.2021.05.014 34049112

[16] Hull NC Schumaker BA. Comparisons of brucellosis between human and veterinary medicine. Infect Ecol Epidemiol. 2018;8(1):1500846. 10.1080/20008686.2018.1500846 30083304 PMC6063340

[17] European Food Safety Authority (EFSA) European Centre for Disease Prevention and Control (ECDC). The European Union One Health 2022 Zoonoses Report. EFSA J. 2023;21(12):e8442. 10.2903/j.efsa.2023.8442 38089471 PMC10714251

[18] Garofolo G Di Giannatale E De Massis F Zilli K Ancora M Cammà C Investigating genetic diversity of Brucella abortus and Brucella melitensis in Italy with MLVA-16. Infect Genet Evol. 2013;19:59-70. 10.1016/j.meegid.2013.06.021 23831636

[19] Vergnaud G Hauck Y Christiany D Daoud B Pourcel C Jacques I Genotypic expansion within the population structure of classical Brucella species revealed by MLVA16 typing of 1404 Brucella isolates from different animal and geographic origins, 1974-2006. Front Microbiol. 2018;9:1545. 10.3389/fmicb.2018.01545 30050522 PMC6052141

[20] Akar K Erganis O. Evaluation of the genetic profiles of Brucella melitensis strain from Turkey using multilocus variable number tandem repeat analysis (MLVA) and multilocus sequence typing (MLST) techniques. Vet Microbiol. 2022;269:109423. 10.1016/j.vetmic.2022.109423 35462118

[21] Scholz HC Vergnaud G. Molecular characterisation of Brucella species. Rev Sci Tech. 2013;32(1):149-62. 10.20506/rst.32.1.2189 23837373

[22] Foster G Osterman BS Godfroid J Jacques I Cloeckaert A. Brucella ceti sp. nov. and Brucella pinnipedialis sp. nov. for Brucella strains with cetaceans and seals as their preferred hosts. Int J Syst Evol Microbiol. 2007;57(Pt 11):2688-93. 10.1099/ijs.0.65269-0 17978241

[23] Holzer K El-Diasty M Wareth G Abdel-Hamid NH Hamdy MER Moustafa SA Tracking the distribution of Brucella abortus in Egypt based on core genome SNP analysis and in silico MLVA-16. Microorganisms. 2021;9(9):1942. 10.3390/microorganisms9091942 34576838 PMC8469952

[24] Brangsch H Sandalakis V Babetsa M Boukouvala E Ntoula A Makridaki E Genotype diversity of brucellosis agents isolated from humans and animals in Greece based on whole-genome sequencing. BMC Infect Dis. 2023;23(1):529. 10.1186/s12879-023-08518-z 37580676 PMC10426126

[25] Akar K Erganis O. Evaluation of the genetic profiles of Brucella melitensis strain from Turkey using multilocus variable number tandem repeat analysis (MLVA) and multilocus sequence typing (MLST) techniques. Vet Microbiol. 2022;269:109423. 10.1016/j.vetmic.2022.109423 35462118

[26] Alton GG, Jones LM, Angus R, Verger J. Techniques for the brucellosis laboratory. Paris: Institut National de la Recherche Agronomique (INRA); 1988.

[27] Bricker BJ Halling SM. Enhancement of the Brucella AMOS PCR assay for differentiation of Brucella abortus vaccine strains S19 and RB51. J Clin Microbiol. 1995;33(6):1640-2. 10.1128/jcm.33.6.1640-1642.1995 7650203 PMC228233

[28] Bankevich A Nurk S Antipov D Gurevich AA Dvorkin M Kulikov AS SPAdes: a new genome assembly algorithm and its applications to single-cell sequencing. J Comput Biol. 2012;19(5):455-77. 10.1089/cmb.2012.0021 22506599 PMC3342519

[29] Wood DE Lu J Langmead B. Improved metagenomic analysis with Kraken 2. Genome Biol. 2019;20(1):257. 10.1186/s13059-019-1891-0 31779668 PMC6883579

[30] Gurevich A Saveliev V Vyahhi N Tesler G. QUAST: quality assessment tool for genome assemblies. Bioinformatics. 2013;29(8):1072-5. 10.1093/bioinformatics/btt086 23422339 PMC3624806

[31] Whatmore AM Perrett LL MacMillan AP. Characterisation of the genetic diversity of Brucella by multilocus sequencing. BMC Microbiol. 2007;7(1):34. 10.1186/1471-2180-7-34 17448232 PMC1877810

[32] Jolley KA Bray JE Maiden MCJ. Open-access bacterial population genomics: BIGSdb software, the PubMLST.org website and their applications. Wellcome Open Res. 2018;3:124. 10.12688/wellcomeopenres.14826.1 30345391 PMC6192448

[33] Stamatakis A. RAxML version 8: a tool for phylogenetic analysis and post-analysis of large phylogenies. Bioinformatics. 2014;30(9):1312-3. 10.1093/bioinformatics/btu033 24451623 PMC3998144

[34] Tan KK Tan YC Chang LY Lee KW Nore SS Yee WY Full genome SNP-based phylogenetic analysis reveals the origin and global spread of Brucella melitensis. BMC Genomics. 2015;16(1):93. 10.1186/s12864-015-1294-x 25888205 PMC4409723

[35] Argimón S Abudahab K Goater RJE Fedosejev A Bhai J Glasner C Microreact: visualizing and sharing data for genomic epidemiology and phylogeography. Microb Genom. 2016;2(11):e000093. 10.1099/mgen.0.000093 28348833 PMC5320705

[36] Akar K Tatar F Schmoock G Wareth G Neubauer H Erganiş O. Tracking the diversity and Mediterranean lineage of Brucella melitensis isolates from different animal species in Turkey using MLVA-16 genotyping. Ger J Vet Res. 2022;2(1):25-30. 10.51585/gjvr.2022.1.0037

[37] Pelerito A Nunes A Grilo T Isidro J Silva C Ferreira AC Genetic characterization of Brucella spp.: whole genome sequencing-based approach for the determination of multiple locus variable number tandem repeat profiles. Front Microbiol. 2021;12:740068. 10.3389/fmicb.2021.740068 34867857 PMC8633399

[38] Pearson T Busch JD Ravel J Read TD Rhoton SD U’Ren JM Phylogenetic discovery bias in Bacillus anthracis using single-nucleotide polymorphisms from whole-genome sequencing. Proc Natl Acad Sci USA. 2004;101(37):13536-41. 10.1073/pnas.0403844101 15347815 PMC518758

[39] Uysal Y. Field experience with Rev.1 vaccine in Turkey. Alfort: FAO/WHO/OIE Round Table on the use of Rev.1 Vaccine in Small Ruminants and Cattle; 1995. Available from: https://www.fao.org/4/ai494e/ai494e00.pdf

[40] Blasco JM Moreno E Moriyón I. Efficacy of Brucella abortus S19 and RB51 vaccine strains: A systematic review and meta-analysis. Transbound Emerg Dis. 2022;69(4):1670-3. 10.1111/tbed.14440 34964556

[41] Daskiran I Savas T Koyuncu M Koluman N Keskin M Esenbuga N Goat production systems of Turkey: nomadic to industrial. Small Rumin Res. 2018;163:15-20. 10.1016/j.smallrumres.2017.10.001

[42] Gürbilek SE Baklan EA Sağlam G Karagül MS Saytekin AM. Conventional and molecular identification of Brucella isolates from livestock in Turkey. Ankara Univ Vet Fak Derg. 2022;69(3):297-302. 10.33988/auvfd.796785

[43] Wareth G El-Diasty M Melzer F Schmoock G Moustafa SA El-Beskawy M MLVA-16 Genotyping of Brucella abortus and Brucella melitensis isolates from different animal species in Egypt: geographical relatedness and the Mediterranean lineage. Pathogens. 2020;9(6):498. 10.3390/pathogens9060498 32580472 PMC7350383

[44] İkikat Tümer E Ağır HB Aydoğan İ. Evaluating technical efficiency of hair goat farms in Turkey: the case of Mersin Province. Trop Anim Health Prod. 2020;52(6):3707-12. 10.1007/s11250-020-02407-2 33026611

[45] Akbay C Akdoğan F. Structure of dairy cattle holdings and market supply of milk: the case of İzmir Province, Turkey. Turk J Agric Res. 2020;7(3):287-95.

[46] Tuncay RM Sancak YC. Presence of Listeria monocytogenes in herby cheese and determination of their susceptibility to antibiotics. Van Vet J. 2018;29(3):169-73.

[47] Altun SK Yiğin A Gürbilek SE Gürbüz S Demirci M Keskin O An enzyme-linked immunosorbent assay for Brucella specific antibody and real-time PCR for detecting Brucella spp. in milk and cheese in Şanliurfa, Turkey. Pak Vet J. 2017;37(1):39-42.

[48] Taha H Smith C Durham J Reid S. Identification of a One Health intervention for brucellosis in Jordan using system dynamics modelling. Systems (Basel). 2023;11(11):542. 10.3390/systems11110542

[49] Gurbilek SE Karagul MS Saytekin AM Baklan EA Saglam G. Investigating field efficacy and safety of conjunctival Brucella abortus S19 vaccine in cattle. Agric Sci Dig. 2022;43(4):556-61. 10.18805/ag.DF-393

[50] Gurbilek SE Karagul MS Saytekin AM Baklan EA Saglam G. Investıgatıng the serologıcal response and safety of Brucella melıtensıs rev.1 conjunctıval vaccıne ın small rumınants. Slovak J Anim Sci. 2023;56(1):30-7. 10.36547/sjas.793

[51] Hikal AF Wareth G Khan A. Brucellosis: why is it eradicated from domestic livestock in the United States but not in the Nile River Basin countries? Ger J Microbiol. 2023;3(2):19-25. 10.51585/gjm.2023.2.0026

[52] Cekanac R Mladenović J Ristanović E Lazić S. Epidemiological characteristics of brucellosis in Serbia, 1980-2008. Croat Med J. 2010;51(4):337-44. 10.3325/cmj.2010.51.337 20718087 PMC2931439

[53] Guler S Kokoglu OF Ucmak H Gul M Ozden S Ozkan F. Human brucellosis in Turkey: different clinical presentations. J Infect Dev Ctries. 2014;8(5):581-8. 10.3855/jidc.3510 24820461

[54] Bingöl KK. İstanbul ve Şanlıurfa'da satışa sunulan urfa peynirlerinin koagülaz pozitif staphylococcus aureus yönüyle karşılaştırılması. [Comparison of Urfa cheeses sold in Istanbul and Şanlıurfa in terms of coagulase positive Staphylococcus aureus]. Istanbul: İstanbul Medipol Üniversitesi Sağlık Bilimleri Enstitüsü; 2016.Turkish. Available from: https://acikerisim.medipol.edu.tr/xmlui/handle/20.500.12511/6859

[55] Namiduru M Gungor K Dikensoy O Baydar I Ekinci E Karaoglan I Epidemiological, clinical and laboratory features of brucellosis: a prospective evaluation of 120 adult patients. Int J Clin Pract. 2003;57(1):20-4. 10.1111/j.1742-1241.2003.tb11391.x 12587937

[56] Tanir G Tufekci SB Tuygun N. Presentation, complications, and treatment outcome of brucellosis in Turkish children. Pediatr Int. 2009;51(1):114-9. 10.1111/j.1442-200X.2008.02661.x 19371290

